# P62 plasmid can alleviate diet-induced obesity and metabolic dysfunctions

**DOI:** 10.18632/oncotarget.19840

**Published:** 2017-08-03

**Authors:** Tatiana Halenova, Oleksii Savchuk, Ludmila Ostapchenko, Andrey Chursov, Nathan Fridlyand, Andrey B. Komissarov, Franco Venanzi, Sergey I. Kolesnikov, Albert A. Sufianov, Michael Y. Sherman, Vladimir L. Gabai, Alexander M. Shneider

**Affiliations:** ^1^ Educational and Scientific Center ‘Institute of Biology and Medicine’, Taras Shevchenko National University of Kyiv, Kyiv, Ukraine; ^2^ Curelab Oncology Inc, Dedham, MA, USA; ^3^ School of Biosciences, University of Camerino, Camerino, Italy; ^4^ Research Institute of Influenza, St-Petersburg, Russia; ^5^ Russian Academy of Sciences, Moscow, Russia; ^6^ Lomonosov Moscow State University, Moscow, Russia; ^7^ Research Center of Family Health and Reproduction Problems, Irkutsk, Russia; ^8^ Federal Center of Neurosurgery, Tyumen, Russia; ^9^ Sechenov First Moscow State Medical University, Moscow, Russia; ^10^ Department of Biochemistry, Boston University School of Medicine, Boston, MA, USA; ^11^ Department of Molecular Biology, Ariel University, Ariel, Israel

**Keywords:** diabetes, high-calorie diet, inflammation, cytokines, serotonin

## Abstract

A high-calorie diet (HCD) induces two mutually exacerbating effects contributing to diet-induced obesity (DIO): impaired glucose metabolism and increased food consumption. A link between the metabolic and behavioral manifestations is not well understood yet. We hypothesized that chronic inflammation induced by HCD plays a key role in linking together the two components of diet-induced pathology. Based on this hypothesis, we tested if a plasmid (DNA vaccine) encoding p62 (SQSTM1) would alleviate DIO including its metabolic and/or food consumption abnormalities. Previously we reported that injections of the p62 plasmid reduce chronic inflammation during ovariectomy-induced osteoporosis. Here we found that the p62 plasmid reduced levels of pro-inflammatory cytokines IL-1β, IL-12, and INFγ and increased levels of anti-inflammatory cytokines IL-4, IL-10 and TGFβ in HCD-fed animals. Due to this anti-inflammatory response, we further tested whether the plasmid can alleviate HCD-induced obesity and associated metabolic and feeding impairments. Indeed, p62 plasmid significantly reversed effects of HCD on the body mass index (BMI), levels of glucose, insulin and glycosylated hemoglobin (HbA1c). Furthermore, p62 plasmid partially restored levels of the satiety hormone, serotonin, and tryptophan, simultaneously reducing activity of monoamine oxidase (MAO) in the brain affected by the HCD. Finally, the plasmid partially reversed increased food consumption caused by HCD. Therefore, the administering of p62 plasmid alleviates both metabolic and behavioral components of HCD-induced obesity.

## INTRODUCTION

Obesity is regarded as one of the main medical problem of the XXI century: according to WHO data (2014), more than 1.9 billion people are overweight, and 600 million are affected by obesity. In the US, obesity rate is currently 35%, compared to 15% in the early 80-s [1]. Obesity often leads to the development of type 2 diabetes (T2D) and comorbid diseases (metabolic syndrome) that provoke early disability and mortality. Despite numerous efforts, however, there is no real cure for obesity and other related diseases.

Experimental and clinical studies of recent years found that obesity is associated with accumulation of macrophages (MF) and chronic inflammation of adipose tissue (AT) which affects its metabolic and secretory functions and plays a key role in obesity-related pathology [2, 3]. There are two major phenotypes of MF: pro-inflammatory, i.e. classically activated (M1) and anti-inflammatory, i.e. alternatively activated (M2). In animals feed with high-calorie diet, there is an increase both in the total number of MF in AT and sharp increase in fraction of M1-MF [4]; accordingly, M1-MF also dominate in AT of patients with obesity or T2D [5]. Importantly, removal of M1-MF prevents development of diet-induced obesity [6, 7].

Chronic inflammation also has a central effect, in particular, by affecting serotonin levels whose release in the brain is among key factors in regulation of appetite [8] [9–11]. Decrease in the content of central serotonin under these conditions can lead to higher food consumption with subsequent hypersecretion of insulin, resulting in insulin resistance and obesity [11, 12].

We have recently developed p62 (SQSTM1)-encoding plasmid (DNA vaccine) which demonstrated anti-cancer activity in animals models [13–15] and in the phase I/IIa clinical trial in patients with solid tumors [16]. p62 is a multifunctional protein which participates in selective autophagy and serves as a signaling hub for several signal transduction pathways, among them NF-kB, TRAF6, MAP kinases, and NRF2 [17] [18, 19] [20] [21]. Various tissues (including AT) in obese animals and humans express less p62 than in non-obese [22] [23]. Furthermore, knockout of p62 led to mature-onset obesity and impaired glucose homeostasis in mice [24], and such effect of p62 knockout was apparently associated with increased activity of ERK in AT and fibroblasts [25]. Of note, knockout of p62 in MF promoted IL-1β accumulation caused by inflammasome activation [26].

We uncovered that the p62 plasmid, but not the empty vector, suppresses chronic inflammation during ovariectomy-induced osteoporosis in mice [27]. Since chronic inflammation is believed to contribute to the development of obesity, we hypothesized that the p62 plasmid can alleviate it as well as related systemic and brain metabolic impairments. Here we tested this hypothesis using a rat model of the diet-induced obesity.

## RESULTS

### p62 plasmid suppresses inflammatory cytokines induced by HCD

Obesity is considered to be a chronic and systemic inflammatory disease where AT overloaded with nutrients plays an important endocrine role through the production of adipokines [2]. Adipokines may activate innate immunity and release of pro-inflammatory cytokines. This, in turn, can induce systemic inflammation that can cause insulin resistance and other metabolic syndromes. Considering beneficial effects of the p62 plasmid on ovariectomy-induced chronic inflammation [27], we explored whether this vaccine can alleviate inflammation in diet-induced obesity (DIO) model which is a common model for study of metabolic diseases. In our model, rats were fed with high calorie diet (HCD) which contained about 40% fat and almost 2-times more calories than standard diet (SD) (28.71 kJ/g vs 15.27 kJ/g). As we found previously, such diet profoundly affected glucose metabolism and led to pre-diabetic state [28].

To test the effect of the vaccine, rats fed with HCD were treated i.m. weekly for 6 weeks or left untreated whereas rats fed with SD served as control. In the end of the experiment (2 weeks after the last 6-th injection of the plasmid), the animals were sacrificed and their sera were collected for analysis of cytokines and other parameters.

**Figure 1:**
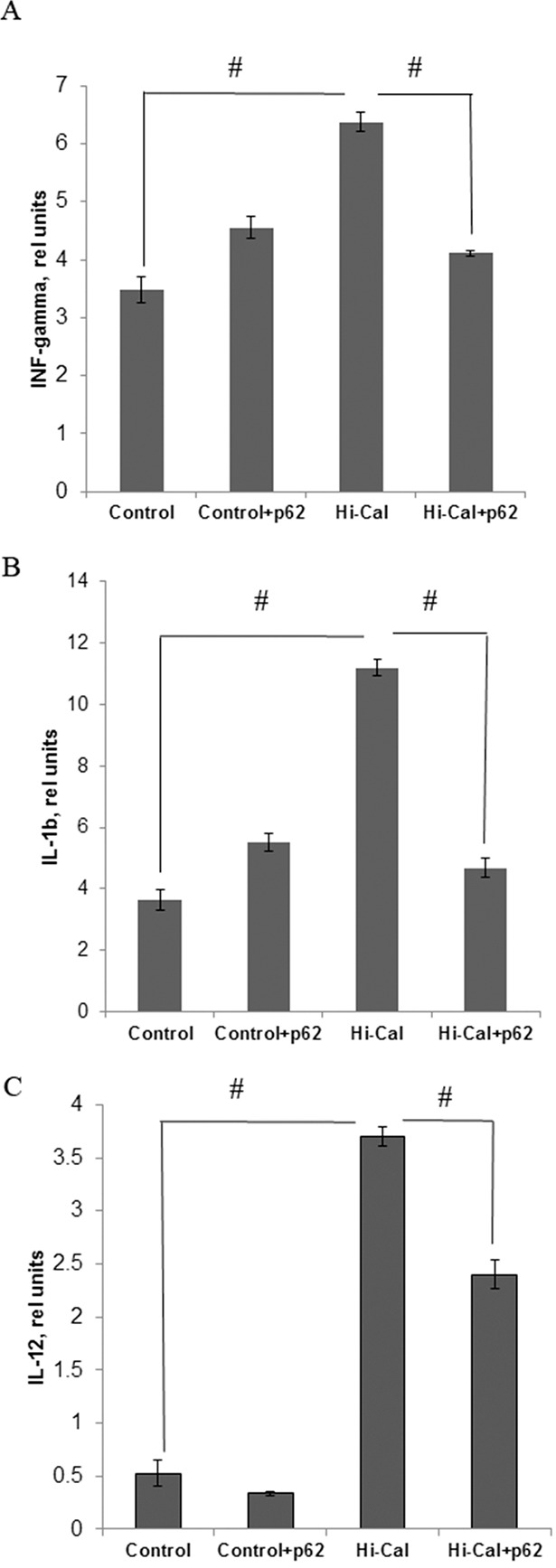
Effect of p62 plasmid on inflammatory cytokines in serum of rats fed with high calorie diet Rats fed with standard diet (control), or high calorie diet (Hi-Cal) were treated i.m. with p62 plasmid 6 times weekly and their sera were collected on 11^th^ week since the start of the experiment. Data shown are means+/−SEM. **(A)** Effect of p62 plasmid on interferon-γ levels. ^#^p= 3.84e-05 Hi-Cal vs control, p=0.00181 Hi-Cal +p62 vs Hi-Cal **(B)** Effect of p62 plasmid on IL-1β levels. ^#^p= 7.34e-05 Hi-Cal vs control, p=0.00201 Hi-Cal +p62 vs Hi-Cal **(C)** Effect of p62 plasmid on IL-12 levels. ^#^p= 3.58e-07 HCD vs control, p= 0.000309 Hi-Cal +p62 vs Hi-Cal.

**Figure 2:**
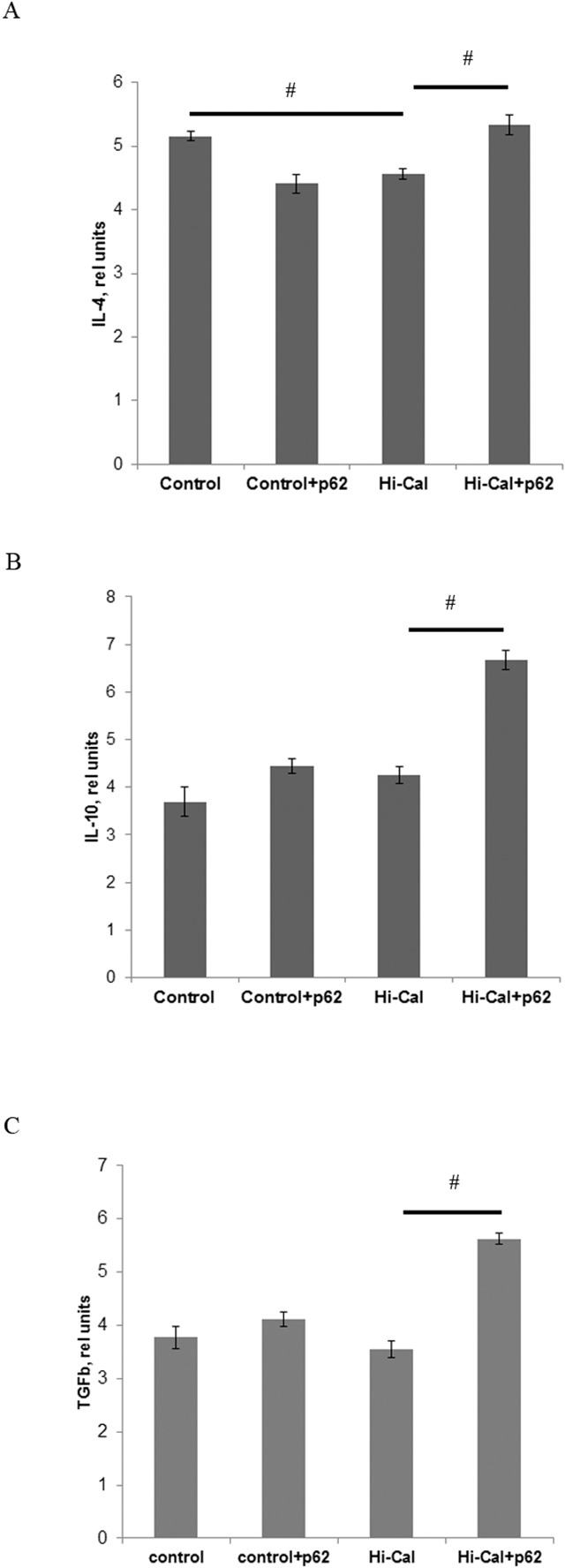
Effect of p62 plasmid anti-inflammatory cytokines in the serum of rats fed with high calorie diet Rats fed with standard diet (control), or high calorie diet (Hi-Cal) were treated i.m. with p62 plasmid 6 times weekly and their sera were collected on 11^th^ week since the start of the experiment. Data shown are means+/−SEM. **(A)** Effect of p62 plasmid on IL-4 levels. # p = 0.012 Hi-Cal vs control, Hi-Cal+p62 vs Hi-Cal **(B)** Effect of p62 plasmid on IL-10 levels. # p = 0.012 Hi-Cal+p62 vs Hi-Cal **(C)** Effect of p62 plasmid on TGFβ levels. # p = 0.012 Hi-Cal+p62 vs Hi-Cal.

We found a marked increase in the levels of pro-inflammatory cytokines in HCD-fed rats comparing to SD-fed (control) rats: 1.83 fold in IFN-γ, 3.1-fold in IL-1β, and 7-fold in IL-12 (Figure 1A-1C). Treatment of HCD-fed rats with the p62 plasmid led to a significantly lower production of all these cytokines: IFN-γ was reduced by 36%, IL-1β - 52%, and IL-12 – 35% compared with the levels of HCD-fed untreated animals (Figure 1A-1C). Thus, the p62 plasmid partially normalized the levels of pro-inflammatory cytokines increased by HCD. As to anti-inflammatory cytokines, there were no significant changes in IL-10 and TGFβ, but IL-4 levels were decreased by 12% in the HCD group compared to the SD group (Figure 2). Treatment of the HCD group with p62 plasmid increased levels of anti-inflammatory cytokines: IL-4 by 16%, IL-10 - 57%, and TGFβ- 58% (Figure 2). Such increased levels of anti-inflammatory cytokines in p62-treated animals fed with HCD may contribute to mitigation of chronic inflammation. Thus, the p62-plasmid may alleviate diet-induced inflammation by both suppression of generation of pro-inflammatory cytokines and augmentation of anti-inflammatory cytokines levels (Figures 1, 2).

### p62 plasmid alleviates high calorie diet induced-obesity and normalizes glucose homeostasis

**Figure 3:**
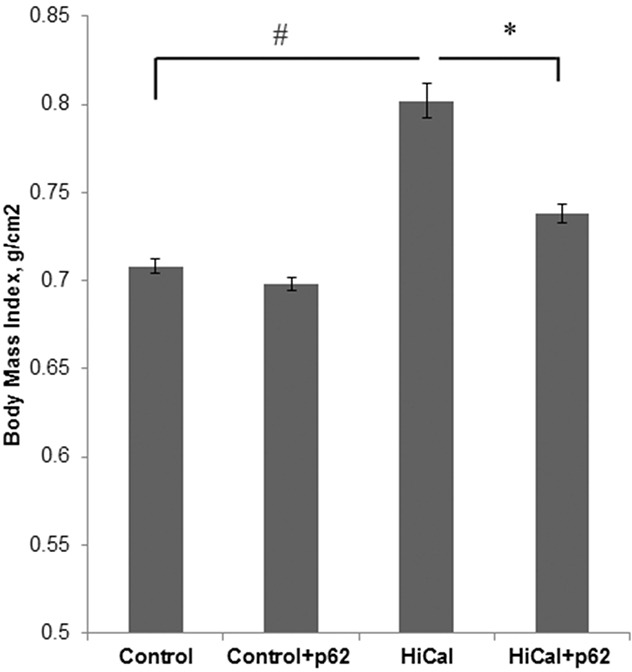
Effect of p62 plasmid on body mass index of rats fed with high-calorie diet Rats fed with standard diet (control), or high calorie diet (Hi-Cal) were treated with p62 plasmid as in Figure 1, 2 and their body mass index was measured on 11^th^ week since the start of the experiment.^#^p= 2.82e-06 Hi-Cal vs control; *p=0.00115 Hi-Cal+p62 vs Hi-Cal.

Based on the effects of the p62 plasmid on chronic inflammation, we sought to test whether it can prevent the development of obesity and alleviate obesity-associated metabolic disorder in our DIO model. To assess the development of the disease, we monitored body mass index (BMI), which reflects changes in body composition of experimental animals and extent of the deposition of fat mass. We found significant increase of BMI in the HCD group compared to the control group (from 0.71 to 0.80) that indicates the development of diet-induced obesity (Figure 3). Administration of the p62 plasmid resulted in a decrease of this parameter to 0.74. In SD-fed animals, the p62 plasmid did not show significant changes in BMI compared to control (Figure 3). Therefore, the p62 plasmid can partially alleviate obesity caused by HCD.

**Figure 4:**
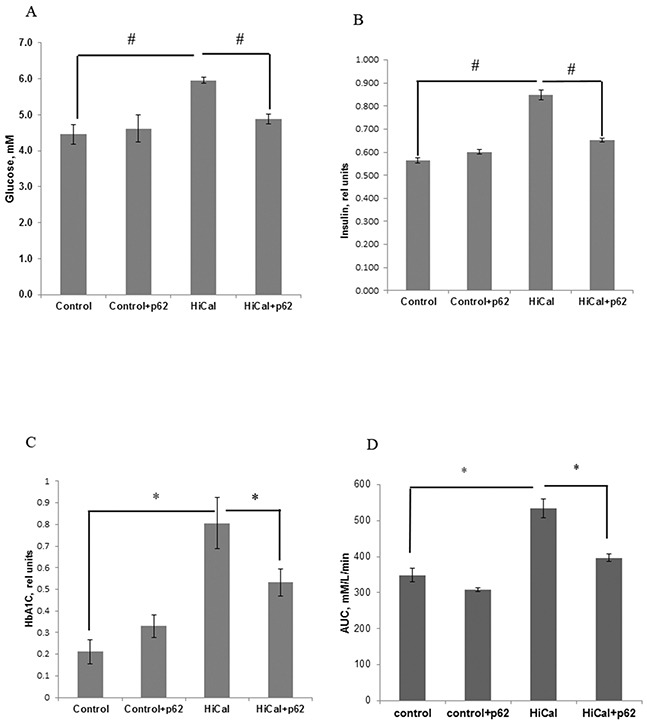
Effect of p62 plasmid on glucose homeostasis in rats fed with high calorie diet Rats fed with standard diet (control), or high calorie diet (Hi-Cal) were treated with p62 plasmid as in Figure 1, 2. Data shown are means+/−SEM. **(A)** p62 plasmid reduces Hi-Cal-induced increase of glucose level. ^#^p= 2.62e-06 Hi-Cal vs control, p= 0.000692 Hi-Cal +p62 vs Hi-Cal **(B)** p62 plasmid reduces Hi-Cal-induced increase of insulin level. #p= 2.46e-07 Hi-Cal vs control, p=0.000231 Hi-Cal +p62 vs Hi-Cal **(C)** p62 plasmid reduces Hi-Cal -induced increase of accumulation of HbA1c. *p= 3.57e-11 Hi-Cal vs control, p= 2.59e-06 Hi-Cal +p62 vs Hi-Cal. **(D)** p62 plasmid decreases Hi-Cal-induced glucose intolerance. Oral glucose tolerance test was performed as described in Materials and Methods. Area under the curve (AUC) between 30 and 150 min was calculated from the data of Supplementary Figure 1 according to formula: AUC_30-150_=1/2(Glucose_30_ + Glucose_150_)x(150-30). *P<0.02 Hi-Cal vs control, Hi-Cal +p62 vs Hi-Cal by Student t-test.

Obesity usually leads to a pre-diabetic state, manifested, in particular, in insulin-resistance and impairment of glucose metabolism. Indeed, as depicted in Figure 4A, 4B, HCD increased insulin and glucose levels as well as the levels of HbA1c (Figure 4C). Additionally, in an oral glucose tolerance test, there is a clear decrease in glucose consumption in HCD-fed rats (Figure 4D, Supplementary Figure 1). Thus, all these parameters demonstrate the development of the pre-diabetic state. Treatment with the p62 plasmid led to a significant reversion of effects of HCD on serum insulin, glucose, and HbA1c levels: the concentration of glucose decreased by 22%, insulin - 30% (Figure 4A, 4B), and HbA1c - 34% (Figure 4C). Also, the p62 plasmid partially reversed glucose intolerance in HCD rats (Figure 4D, Supplementary Figure 1).

### p62 plasmid increases brain serotonin and tryptophan levels in rats fed with HCD

**Figure 5:**
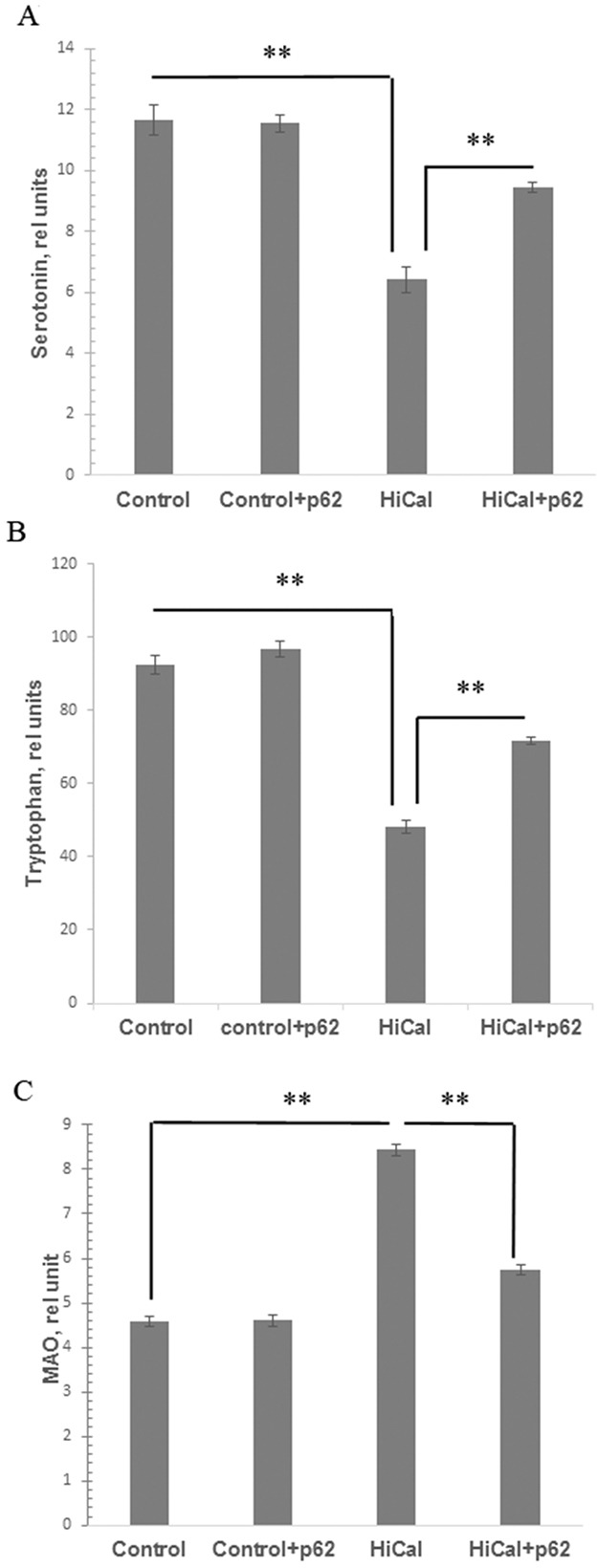
Effect of p62 plasmid on serotonin, tryptophan, and MAO in brain Rats fed with standard diet (control), or high calorie diet (Hi-Cal) were treated with p62 plasmid as in Figure 1, 2, and their brain homogenates were prepared on 11^th^ week since the start of the experiment. Data shown are means+/−SEM. **(A)** Effect of p62 plasmid on serotonin levels. **p= 5.28e-11 Hi-Cal vs control, p=5.53e-06 Hi-Cal +p62 vs Hi-Cal **(B)** Effect of p62 plasmid on tryptophan levels. **p= 1.58e-10 Hi-Cal vs control, p= 4.04e-06 Hi-Cal +p62 vs Hi-Cal **(C)** Effect of p62 plasmid on MAO activity. **p= 1.51e-10 Hi-Cal vs control, p=3.9e-06 Hi-Cal +p62 vs Hi-Cal

The metabolism of brain serotonin, one of the key factors in feeling satiety, can be affected by obesity [10, 11]. Indeed, we found a significant decrease in the levels of serotonin and its metabolic precursor tryptophan in the brains of HCD-fed rats (1.8 times and 1.9 times, respectively) (Figure 5A, 5B). Treatment of the HCD-fed rats with the p62 plasmid led to a partial restoration of these parameters (47% increase for serotonin, and 48% - for tryptophan levels, Figure 5A, 5B). Activity of MAO (monoamine oxidase) involved in the degradation of serotonin increased almost 2-fold in obese rats but the p62 plasmid significantly (by 32%) suppressed MAO activity (Figure 5C). These results show a positive effect of the vaccine on serotonin metabolism in the brain of rats during DIO. We then analyzed the data on food (calories) consumption in the rats feed with HCD. As expected, these rats consumed many more calories than rats fed with a standard diet (Supplementary Figure 2). However, the p62 plasmid treatment normalized calorie consumption in DIO rats (Supplementary Figure 2)

## DISCUSSION

Obesity leads to many dysfunctions: the imbalance in glucose metabolism observed in patients with obesity can provoke the development of type 2 diabetes (T2D), whereas the imbalance in the protein and lipid metabolism can lead to cardiovascular diseases. Therefore, the prevention and reversal of obesity is an urgent medical problem. Here we tested a novel approach to treat obesity: by weekly intramuscular administration of the plasmid (DNA vaccine) encoding p62 (SQSTM1) protein. In the DIO rat model this treatment decreased systemic generation of inflammatory cytokines, normalized glucose homeostasis and alleviated obesity (Figures 1–4). The finding expands our previous observation that the p62 plasmid can alleviate chronic inflammation and bone pathology in rodent models of ovariectomy-induced osteoporosis [27].

The question arises of how a rather small amount (200 ug) of locally injected plasmid may have a systemic effect (on cytokines in blood), and remote effects, in particular, on AT and brain (i.e., on its serotonin metabolism). One of possibilities is that p62 plasmid acts as a plain DNA vaccine by generating immune response to the encoded antigen, p62, and thus damaging target tissue(s) where p62 is overexpresed (e.g. in tumors) [13, 15, 29]. However, tissues including AT in obese animals express less p62 than in non-obese [22] [23], and p62 knockout increases rather than decreases inflammation [30], therefore this possibility looks unlikely.

Instead, we suggest that the p62-encoding plasmid reduces chronic inflammation via modulation of the macrophage (MF) state by switching M1/M2 balance toward M2. This hypothesis is based on several sets of data: 1) proinflammatory M1-MF accumulate in AT during obesity, while their transformation to M2-MF aleviate obesity [4, 5, 31];2) plasmids can rather easily penetrate to bone marrow upon i.m. injection [32] [33] where AT-associated MF are generated from monocytes [34]; 3) monocytes (M0) and MF, in particular, M2-MF, can migrate from bone marrow to the sites of inflammation [35, 36]; 4) p62 overexpression in MF can inhibit their proinflammatory state (i.e. generation of proinflammatory cytokines) [37]. Thus, the administered p62 plasmid goes from the injection site to the bone marrow where it can transform M0 and/or M1-MF shifting them to M2-MF; then these M2-MF migrate to different tissues including AT where they alleviate inflammation, obesity and related pathologies.

**Figure 6:**
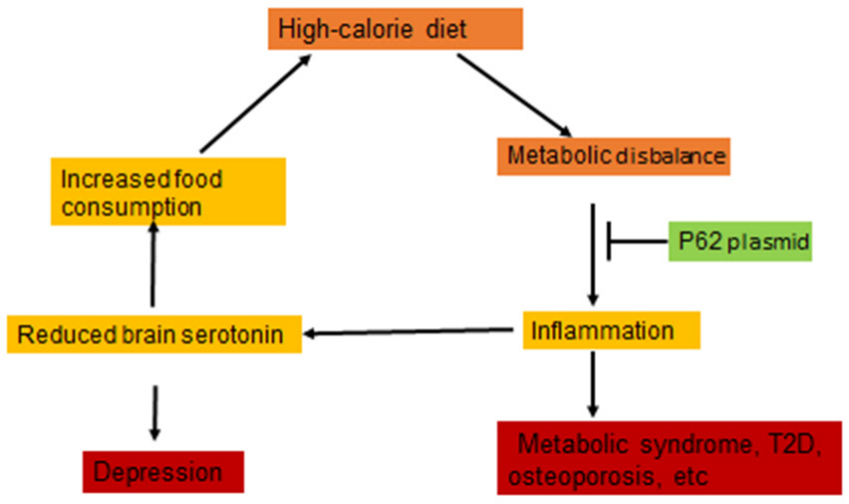
p62 plasmid disrupts “vicious circle” of diet-induced obesity Metabolic disbalance induced by high calorie diet causes inflammation which can lead to reduced serotonin in the brain. This, in turn, can increase food consumption thus causing “vicious circle”. p62 plasmid apparently disrupts this circle by suppressing inflammation.

We observed that HCD caused dysfunction of the serotonin pathway of tryptophan metabolism in the brain of rats: a decrease in the content of tryptophan and serotonin (Figure 5). Serotonin is a satiety hormone; reduced serotonin levels can lead to disruption of the serotonin-dependent regulation of feelings of satiety and the development of hyperphagia in obesity [10–12] [38]. Also, we recorded increased activity of monoamine oxidase, an enzyme which metabolizes and reduces serotonin levels, in the brain of rats of DIO group (Figure 5). The excessive uptake of nutrients from food, particularly free amino acids, may lead to an imbalance in the central dopamine and serotonin systems, which causes development of hyperphagia thus creating a positive feedback loop between mental and metabolic dysfunctions (Figure 6). Chronic inflammation can be a link forming such “vicious circle” of obesity: HCD consumption disrupts the metabolism; metabolic dysfunction causes chronic inflammation; chronic inflammation leads to a reduced serotonin level, and reduced serotonin level further causes higher HCD consumption o (Figure 6). Indeed, there are several reports that systemic inflammatory cytokines and local MF (microglia) can affect brain serotonin metabolism by decreasing its levels [9] [39].

Dysfunction of the serotonergic system is the basis for the development of a number of pathological conditions and mental disorders possessing comorbidities with metabolic syndromes and T2D, including schizophrenia, various psychoses, anxiety and depression [40]. Depression often increases food consumption which leads to obesity [41]. As we described above, DIO in turn disrupts the serotonin system, which closes the circle: insufficient serotonin level causes depression; depression can lead to the increased calorie consumption; excessive calories cause systemic inflammation, and inflammation can further reduce serotonin levels (Figure 6). Thus, not only the metabolic and behavioral components of DIO can aggravate each other forming a positive feedback loop, but also metabolic and mental disorders may be mutually exacerbating.

We found that the p62 plasmid can apparently break this cycle: in addition to normalizing metabolic parameters it also partially normalized parameters of brain serotonin metabolism impaired by HCD (Figure 5) and reduced calorie uptake (Supplementary Figure 2). We suggest that such increase in brain serotonin levels in DIO rats treated with the p62 plasmid is a secondary effect to the suppression of inflammation. Thus, reducing chronic inflammation, the p62 plasmid can break the “vicious cycle” of obesity alleviating both metabolic and behavioral components of HCD -induced obesity (Figure 6).

To our knowledge, this is the first report that a DNA vaccine can alleviate diet-induced chronic inflammation, metabolic dysfunctions and weight gain. The main focus of future research should be revealing the mechanism by which the p62 plasmid suppresses chronic inflammation that is involved in a range of diseases besides obesity [42, 43]. Also, recent reports about the role of MAO in tumor metastasis [44, 45] make it interesting to test whether inhibition of MAO contributes to an anti-metastatic effect of the p62 plasmid which we observed in mice [13].

## MATERIALS AND METHODS

### Animals and diets

The experiments were conducted in 5-weeks old Wistar male rats with initial weight of 135-140 g. The animals were housed and handled in animal facility after approval of scientific council of T. Shevchenko Kiev National University according to EU Directive 2010/63/EU for animals. The animals of each experimental group were housed in polypropylene cages in an environmentally controlled clean air rooms. They were adapted for a week and feed with a SD (standard diet) consisting of 6.7% fat, 21% protein and 55.1% of carbohydrate (15.27 kJ/g, Laboratory Rodent Diet #5001, LabDiet ) and water ad libitum and then the rats were divided in two matched groups: control (continued to be fed with a SD) and fed with HCD (high calorie diet) consisted of 60% of a SD with 10% of pork fat, 10% eggs, 9% sucrose, 5% peanuts, 5% dry milk, 1% of vegetable oil (38.8% total fat, 28.71 kJ/g) [28]. Calorie consumption was calculated per each cage (5 rats) per day based on daily food consumption.

### p62 plasmid treatment

Human p62 (Sqstm1) – encoding DNA vaccine was described elsewhere [13, 27] and produced using EndoFree Plasmid Giga Kit (Qiagen). Three weeks after feeding with a SD or HCD, half of the animals from each group were randomly selected and subjected to the treatment with the p62 plasmid which was intramuscular injected in femoral quadriceps with 200 ug (100 ul) per rat once a week for 6 weeks; another half did not receive injections. Thus, there were 4 experimental groups: 1) control (SD) -10 animals; 2) control+p62 – 10 animals; 3) HCD – 15 animals; and 4) HCD+p62 plasmid – 15 animals). We did not use empty vector (pcDNA3.1) as a control to save animals since in our previous studies the empty vector, similar to saline, did not have any effects on inflammation [27] or tumor growth [13]. Body mass index (weight (g)/lenght^2^ (cm)) and other parameters were measured at the end of experiment (on 11th week, i.e. 2 weeks after the last 6^th^ injection of p62 plasmid).

### Biochemical analyses

A serum was obtained from tail vein and assessed for glucose level via glucose-oxidase method utilizing Glucophot-II glucometer (Ukraine). Animals were fasting for 6 hr prior to the blood sampling. Glycosylated hemoglobin was measured spectrophotometrically with “Pliva-lachema Diagnostika” (Chech Republick) assay kit.

Serum insulin and cytokines (interferon gamma, TGF and interleukins 1-beta, 4, 10 and 12) were quantified by ELISA immune assays with primary and secondary antibodies from Santa Cruz Biotechnology, CA, USA. The data were normalized by serum protein which was measured by Bradford assay.

### Oral glucose tolerance test

Before the test, animals fasting for 6 hr were anesthetized by an i.p. injection of thiopental sodium, at a dose of 40 mg/kg, and glucose (3 g/kg in 2 ml) was administered *per os*. Blood samples (100 ul) were taken via an i.v. catheter from tail vein before the test and at 30, 60, 90, 120, 150 min after the administration of glucose; serum glucose concentration was determined as described above. Based on the test results, the glycemic curve was depicted and area under the curve (AUC) calculated.

### Assay of serotonin, tryptophan and monoamine oxidase

Brain homogenates from fasting for 6 hr and euthanized animals were prepared using homogenization medium containing 50 mM Tris-acetate buffer, pH 7.4, 5 mm EDTA and 10% sucrose with the tissue:buffer ratio of 1:10 (w/w). The homogenates were centrifuged for 20 min at 1500 g. All the procedures were carried out at +4°C. The resulting supernatant was used to measure the concentrations of serotonin, tryptophan and MAO (monoamine oxidase) activity as described before [38]. Briefly, serotonin and tryptophan were quantified using ion-exchange chromatography on KM-Sepharose with subsequent measurement of fluorescence of tryptophan (at excitation at 359 nm and absorption at 485 nm), and serotonin (excitation 295 nm and absorption 550 nm). MAO activity of the brain was assayed using kinuramine dihydrobromide as a substrate by measuring fluorescence of its product, 4-hydroxyquinoline, at excitation at 315 nm and absorption at 380 nm as described [38].

### Statistical analysis

Data shown are means+/−SEM. p-values were calculated using Kruskal-Wallis tests and the successive post hoc Conover-Iman tests if not mentioned otherwise; p<0.05 was considered statistically significant. Analysis was performed using the R software.

## SUPPLEMENTARY MATERIALS FIGURES


