# Supplementary material for: P62 plasmid can alleviate diet-induced obesity and metabolic dysfunctions
Source: Oncotarget. 2017 Aug 3;8(34):56030–40. doi: 10.18632/oncotarget.19840 (PMC5593542; doi:10.18632/oncotarget.19840)
Supplement: Supplementary file 1 [file oncotarget-08-56030-s001.pdf]

P62 plasmid can alleviate diet-induced obesity and metabolic dysfunctions

SUPPLEMENTARY MATERIALS

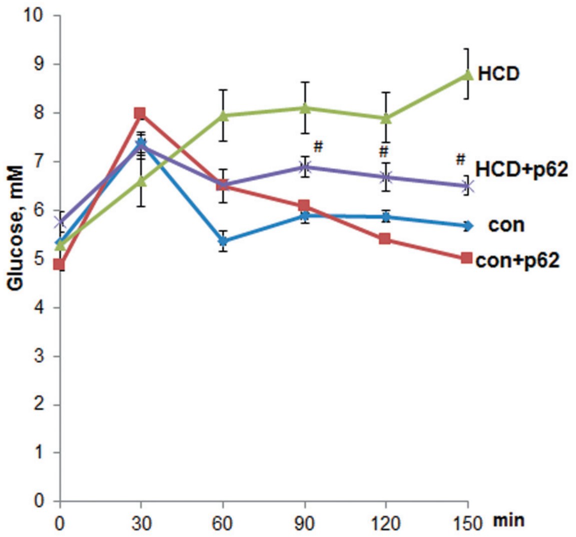

Supplementary Figure 1: p62 plasmid normalizes glucose tolerance in rats fed with high-calorie diet (HCD). Glucose (3 g/kg *per os*) was administered in animals fasting for 6 hr and was assayed in blood at the times indicated. *p<0.001 HCD+p62 vs HCD by Student paired t-test.

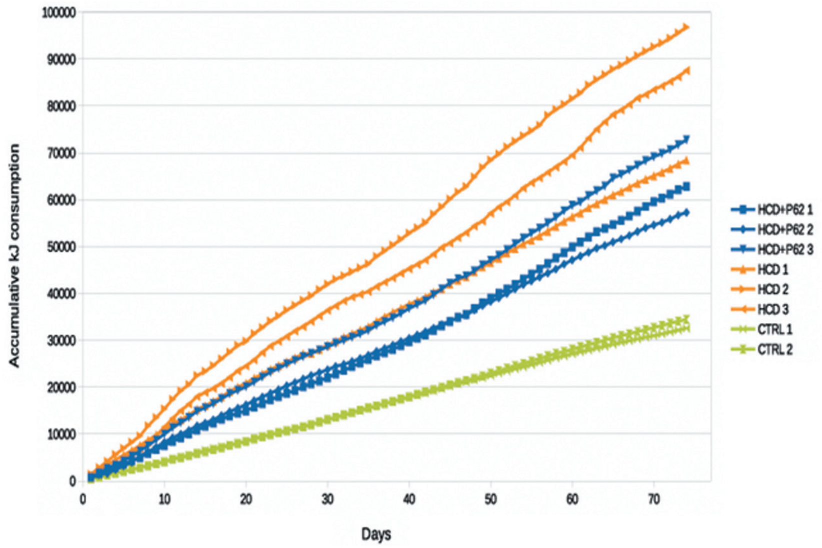

Supplementary Figure 2: Effect of p62 plasmid on calorie consumption of rats fed with high-calorie diet (HCD). Calorie consumption was calculated per each cage (5 rats) per day based on their food consumption; there were 2 cages with control diet; 3 cages with HCD, and 3 cages with HCD+p62. Rats with HCD consumed more calories than control rats, whereas p62 diminished calorie consumption in HCD rats. P62 in control rats does not have any effect and omitted for clarity.
